# Selection of Key Genes for Apricot Kernel Oil Synthesis Based on Transcriptome Analysis

**DOI:** 10.3390/foods14040568

**Published:** 2025-02-08

**Authors:** Dan Zhang, Zhong Zhao

**Affiliations:** 1 College of Forestry, Northwest A&F University, Yangling 712100, China; 13897288320@163.com; 2 Key Laboratory of Silviculture on the Loess Plateau State Forestry Administration, Northwest A&F University, Yangling 712100, China

**Keywords:** apricot, oil, transcriptome, fatty acid composition, WGCNA

## Abstract

The purpose of this study was to identify the key genes regulating apricot kernel oil (AKO) biosynthesis and understand the molecular pathways of AKO synthesis and accumulation. This study used two varieties of apricot kernel to determine the oil contents and primary fatty acid compositions at different developmental stages. Candidate genes for AKO biosynthesis were selected through transcriptome sequencing technology and weighted gene co-expression network analysis (WGCNA), and these genes were verified by quantitative reverse transcription polymerase chain reaction (qRT-PCR). The results indicate that during apricot ripening, the content of AKO exhibits an ‘S’-shaped accumulation pattern. The primary fatty acid components are C18:1 and C18:2. The transcriptome sequencing produced 164.19 Gb of clean data and 17,411 differentially expressed genes. The WGCNA results indicate that significantly differentially expressed genes cluster into seven modules—gene clusters (module)—with the strongest correlations to AKO indicated in pink. Nineteen candidate genes were selected from the oil synthesis pathway and WGCNA results. The qRT-PCR results indicate that six key enzyme genes and three transcription factors play significant regulatory roles in AKO biosynthesis. This study elucidates the molecular pathways involved in AKO biosynthesis and explains the difference in oil content between bitter and sweet apricot kernels.

## 1. Introduction

Apricot (*Prunus armeniaca* L.), an economically significant tree species, is part of the Rosaceae family. Apricots originated in China and are extensively cultivated globally [1,2]. Apricot is one of the important woody oilseed species, with the apricot kernel oil (AKO) content exceeding 50% [3]. More than 90% of the fatty acids in AKO are unsaturated fatty acids such as oleic and linoleic acids, making it a source of high-quality cooking oil and a feedstock for biodiesel [4]. The apricot kernel also has many medical properties such as anticancer, antioxidant, asthma, and anti-microbial activity [5]. Apricot kernels are classified into sweet and bitter varieties. The sweet apricot kernel has a significantly higher oil content than the bitter apricot kernel. Sweet AKO is widely used domestically and internationally in the cosmetics field due to its skincare benefits. Bitter AKO contains a higher level of amygdalin and is widely used in the food field after detoxification processing. Therefore, AKO has excellent potential for high-value processing, and it is important to investigate the mechanism of oil biosynthesis.

The biosynthesis process of plant oil has been reported. Acetyl coenzyme A (Acetyl-CoA) forms free fatty acids, which are catalyzed by long-chain acyl coenzyme A synthetase (LACS) to synthesize fatty acid coenzyme A (FA-CoA) [6]. Acyl-CoA and glycerol-3-phosphate (G3P) combine to form diacylglycerol (DAG) via the “Kennedy pathway” within the endoplasmic reticulum (ER) [7]. Finally, the DAG is esterified by diacylglycerol acyltransferase (DGAT) to produce triacylglycerol (TAG). In addition, there are complex PC pathways for synthesizing TAG in different plants [8]. Eventually, TAG combines with oil body proteins to form oil bodies and is stored in seeds [9].

In recent years, the rapid development of sequencing technology has provided excellent facilities for plant research, and a large amount of genomic and transcriptomic data are obtained by sequencing to discover functional genes. However, the large amounts of data obtained from sequencing require scientific analytical methods to quickly and accurately identify candidate genes needed for research. Weighted gene co-expression network analysis (WGCNA) is a biological analysis method that constructs gene co-expression networks based on the correlation of gene expression patterns. Thresholds are set in co-expression networks to divide closely related gene clusters into different modules, each represented by different colors. Genes within each color (module) have similar functions [10]. Key genes involved in regulation are quickly identified by analyzing the correlation between various modules and specific traits. In apricots, transcriptome sequencing and WGCNA have also been applied in many related studies, such as flavonol biosynthesis [11]; apricot fruit ethylene metabolism and pectinase activity [12]; growth hormone signaling, cell wall loosening, and fruit size regulation [13]; fruit flavor compound metabolism [14]; petal color heterochromatic formation [15]; and anthocyanin accumulation and carotenoid metabolism, among other key genes screened [16]. However, fewer studies related to oil synthesis or accumulation have been reported. Niu [17] analyzed the transcriptome data of the apricot kernel and characterized two transcription factors (*WRI1* and *FUS3*) and five enzyme genes (*ACCase*, *FATB*, *FATA*, *DGAT1*, and *PDAT2*), which demonstrated that these genes play an important regulatory role in AKO accumulation. Dang [18] performed transcriptome sequencing analysis and found that the bilberry oleosomal protein gene *PsOLE4* has seed expression specificity and promotes fatty acid content in transgenic *Arabidopsis thaliana*. Deng [10] used WGCNA to identify 161 WRI1 co-expressed genes, which were mainly involved in carbon allocation and FA biosynthesis, revealing the mechanism of carbon allocation optimization of *WRI1* in the process of lipid accumulation. The reports concerning the synthesis of AKO are insufficient. Figuring out the molecular mechanism of AKO synthesis is an important prerequisite for the high-value processing and utilization of AKO. Oil accumulation is a complex network, and further studies are needed to reveal its molecular mechanism. The analysis above highlights several knowledge gaps and shortcomings that still need to be addressed. The objectives of this study were to (1) measure the oil content and fatty acid composition of two varieties of apricot kernel at different developmental stages; (2) select key candidate genes by transcriptome sequencing technology combined with WGCNA; and (3) analyze the relative expression data of the candidate genes to identify key genes for AKO synthesis.

## 2. Materials and Methods

### 2.1. Experimental Materials

The apricot kernel samples applied in this study were collected from the almond germplasm resource nursery of the Weihe Experimental Station of the Northwest Agriculture and Forestry University. Two varieties of apricot kernel, ‘Wei-Xuan-1’ (WX-1) and ‘Shanku-1’ (SK-1), were selected for the experiment. Flowering branches (more than 50% of the tree’s flower buds) were marked as sampling branches. Sampling was started on the 10th day after flowering (DAF10), and then the sampling was every 10 days until fully mature, with the sampling time was from 9:00 a.m. to 11:00 a.m. The peels of apricot fruits were removed, and the apricot kernel was quickly packed into freezing tubes in liquid nitrogen and stored in an ultra-low-temperature refrigerator for molecular testing of the transcriptome. The apricot kernel used for oil extraction and fatty acid component determination was harvested with the skin and hulls broken and freeze-dried in a freeze-dryer for later use.

### 2.2. Extraction and Sequencing of Apricot Kernel Total RNA

According to the pre-experiment, four different lipid accumulations DAF30, DAF50, DAF70, and DAF90 were selected for transcriptome sequencing in almond samples of two varieties, with three replicates at each stage, totaling 24 samples. In this experiment, total RNA from apricot nuclei was extracted according to the following steps: (1) the sample was ground into a fine powder using liquid nitrogen, (2) the cells were lysed and RNA enzymes were inactivated by lysate, the PLANTaid (Aidlab Biotechnologies Co., Ltd., Beijing, China) was added to remove polysaccharide polyphenols, (3) the genomic DNA was removed using a genomic clearance column, and (4) cell metabolites and impurities such as proteins were removed using a deproteinization solution followed by a rinse solution. Finally, RNA was collected in RNase-free H_2_O (Aidlab Biotechnologies Co., Ltd., Beijing, China). The agarose gel electrophoresis was performed as follows. The 1.0% agarose solution was heated and then poured into the mold where the comb had been inserted. The 1% TAE buffer was added to the electrophoresis tank until the gel was completely submerged. Subsequently, 5 μL dl2000 DNA Marker (Takara Bio, Beijing, China), a mixture of 5 μL RNA samples, and 1 μL 6× Loading Buffer (Takara Bio, Beijing, China) were successively added to the gel sample hole. The voltage and current were set to 150 V and 100 mA, respectively. The agarose gel (Beijing Solarbio Science & Technology Co., Ltd., Beijing, China) was removed after running for 15 min, and the electrophoretogram of total RNA was observed and captured using the gel imaging system. The samples that passed the quality control were entrusted to Wuhan MetWare Biotechnology Co. (Wuhan, China) for transcriptome sequencing analysis.

### 2.3. Splicing Assembly of Apricot Kernel Transcriptome Data

The high-quality total RNA extracted in Section 2.2 was subjected to library construction, and sequencing was performed using the Illumina HiSeq platform after library checking. Clean data were filtered from the raw data, and they were then compared to the apricot reference genome “https://www.ncbi.nlm.nih.gov/genome/11012 (accessed on 12 September 2023)” using HISAT2 after quality control; we obtained positional information on the reference genome and sample-specific sequence characterization information. The comparison efficiency (Mapped Reads as a percentage of Clean Reads) was checked, and we visualized the results. Reads were assembled into transcripts using the String Tie application network streaming algorithm and optional de novo assembly.

### 2.4. Annotation of Gene Function

The assembled transcripts were annotated against NR (NCBI non-redundant protein sequences), KEGG (Kyoto Encyclopedia of Genes and Genomes), TrEMBL annotations, KOG (Gene Ontology) annotations, GO (Gene Ontology), Pfam (protein family), and SwissProt (a manually annotated and reviewed protein sequence database) public databases by the BLAST tool “http://blast.ncbi.nlm.nih.gov/Blast.cgi (accessed on 1 June 2024)”.

### 2.5. Pathway Analysis of Differentially Expressed Genes

FPKM (fragments per kilobase of exon model per million mapped fragments) was used as a measure of the transcript or gene expression level. Unstandardized read count data (implemented by feature counts) were entered into the DESeq2 package of R software for (4.2.2) differential expression gene (DEG) analysis between the two variety groups, and the sets of DEGs in 24 samples were obtained after testing and correction.

Hierarchical clustering analysis of the DEG analysis were performed in R software, and significance enrichment analysis was performed using Pathway in the KEGG database. To construct scale-free networks and delineate correlation modules based on TOM matrix clustering, we used R software and the WGCNA package [19]. The specific modules related to lipid synthesis were identified by KEGG enrichment analysis, and the hub genes were selected and analyzed by Cytoscape (version 3.6.1) software for network visualization.

### 2.6. Determination of AKO Content and Fatty Acid Fractions

The content of oil extracted using petroleum ether (60–90) (Rhawn, Shanghai, China) as a solvent was calculated via Equation (1):

Oil content (%) = (W2 − W3)/W1 × 100%(1)

where W1 represents the weight of kernel powder; W2 represents the weight of dried filter paper wrapped with kernel powder; and W3 represents the weight of filter paper wrapped with kernel powder after 8 h of extraction.

GC chromatographic conditions: the chromatographic column was an HP-88 Agilent (0.20 μm × 0.25 mm × 100 m) capillary column. Four-step heating procedure: (1) 100 °C, 13 min; (2) 180 °C (V = 10 °C/min), 6 min; (3) 192 °C (V = 1.5 °C/min); (4) 240 °C (V = 3.5 °C/min), 4 min. The inlet port was at 250 °C, the fractional flow ratio was 1:20, the carrier gas was N_2_, and the flow rate of the column was 1.0 mL/min. The FAME peaks of fatty acids were obtained by comparing the retention times with the fatty acid mixture standards, and the relative fatty acid percentage (%) was calculated from the peak area.

### 2.7. qRT-PCR of Candidate Genes for AKO Synthesis

According to the transcriptome sequencing results and WGCNA, 19 candidate genes were selected, comprising 14 genes for AKO synthases and 5 genes for transcription factors. The Primer Premier 5 software was used to design quantitative primers for lipid synthesis-related genes (primer sequences are shown in Table 1). The total RNA from the samples was extracted after grinding the apricot kernel into fine powder in liquid nitrogen, as described in Section 2.2. Reverse transcription was carried out following the guidelines for the TAKARA PrimeScriptTM RT Reagent Kit with gDNA Eraser (Perfect Real Time) (Takara Bio, Beijing, China). The cDNA was generated by eliminating genomic DNA and subsequently performing reverse transcription. Based on the literature reports [20], three candidate reference genes were chosen for pre-experimentation to determine the final reference genes, and the relative expression of the candidate genes was detected by the qRT-PCR method.

## 3. Results

### 3.1. Transcriptome Data Analysis and Assembly

**Figure 1:**
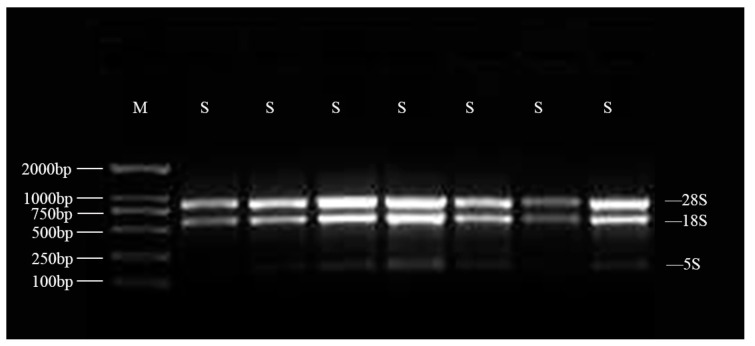
Electrophoretic map of total RNA in partial apricot kernel. Note: M denotes the electrophoretic band of the DL2000 DNA Marker, while S signifies the apricot kernel sample.

The results of RNA agarose gel electrophoresis showed that the 18S and 28S bands were clearly defined without tail dragging (Figure 1). The OD values range from 1.8 to 2.1, which indicated that the total RNA was of high quality and suitable for subsequent library construction and sequencing needs. Transcriptome sequencing showed that 164.19 Gb of clean data was obtained from the cDNA library of 24 samples, with each sample reaching 6 Gb of clean data. The percentage of Q20 bases was 97% and above, the rate of Q30 bases was 92% and above, and the GC content ranged from 45.79% to 51.19%.

After quality control, clean reads were compared to the reference genome. The comparison efficiency of all 24 samples in this test was greater than 90%, and the proportion of the comparison region distributed in the exon region was greater than 57%. This indicated that the reference genes were consistent with the sequenced species, and there was no contamination in the test.

### 3.2. Visualization of Expression Differences

**Figure 2:**
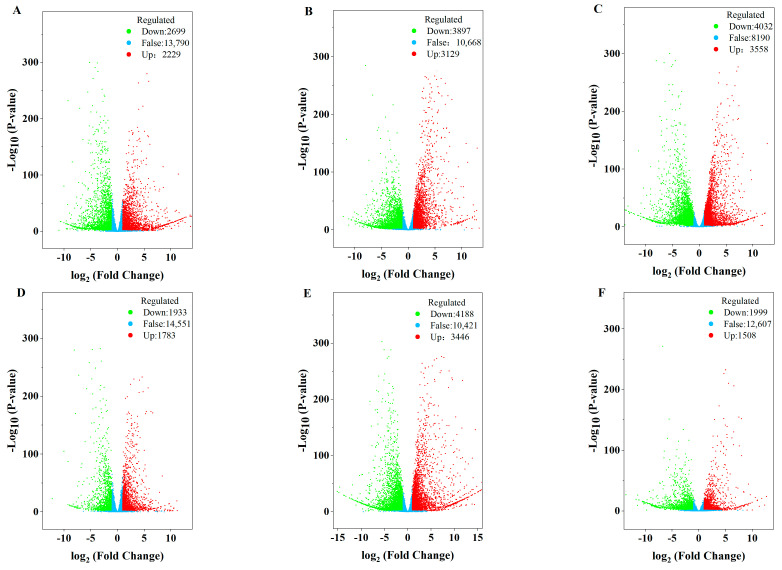

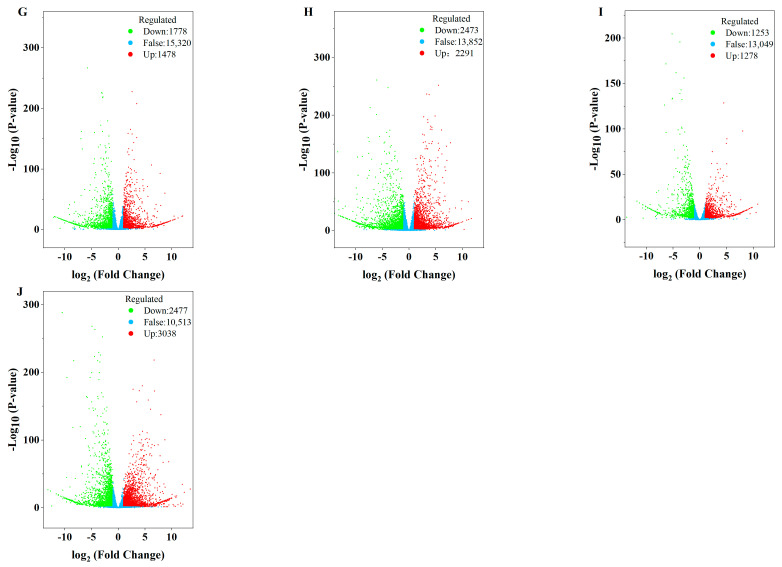
Volcano map of differentially expressed genes. Note: Red represents up-regulated genes, green represents down-regulated genes, and blue represents non-significant genes. (**A**) SK−1 DAF30 vs. SK−1 DAF50, (**B**) SK−1 DAF50 vs. SK−1 DAF70, (**C**) SK−1 DAF70 vs. SK−1 DAF90, (**D**) WX−1 DAF30 vs. WX−1 DAF50, (**E**) WX−1 DAF50 vs. WX−1 DAF70, (**F**) WX−1 DAF70 vs. WX−1 DAF90, (**G**) SK−1 DAF30 vs. WX−1 DAF30, (**H**) SK−1 DAF50 vs. WX−1 DAF50, (**I**) SK−1 DAF70 vs. WX−1 DAF70, (**J**) SK−1 DAF90 vs. WX−1 DAF 90.

Based on the homogenized FPKM values, differential expression gene analysis was performed using DESeq2. The total number of differentially expressed genes was quantified. The results are shown in Figure 2. There were 17,411 differentially expressed genes in the 24 samples. The variation in gene differential expression was specific to each variety. From DAF70 to DAF90, ‘SK-1’ had the most differentially expressed genes (Figure 2C), totaling 7590, with 3558 up-regulated and 4032 down-regulated. ‘WX-1’ had a total of 7634 differentially expressed genes from DAF50 to DAF70 (Figure 2E), with 3446 up-regulated genes and 4188 down-regulated genes. During the period from DAF70 to DAF90 (Figure 2J), the number of differentially expressed genes was highest in the two varieties. This period also coincided with rapid oil accumulation.

### 3.3. WGCNA and Hub Gene Selection

**Figure 3:**
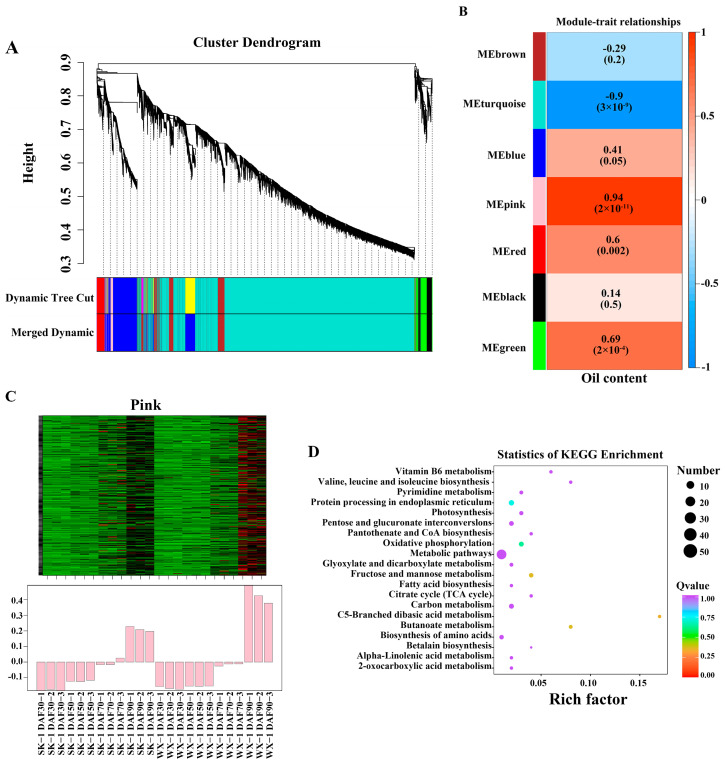
WGCNA cluster analysis. (**A**) Cluster dendrogram; (**B**) module–trait relationships (above is the correlation coefficient, below is the *p*-value); (**C**) module heatmap, Green indicates low expression levels, while red signifies high expression levels; (**D**) KEGG enrichment analysis.

Differentially expressed genes were screened from 24 samples and analyzed by WGCNA. The results showed that the differentially expressed genes were classified into seven different modules, which were represented by brown, turquoise, blue, pink, red, black, and green, respectively (Figure 3A). The size of the color block area indicates the number of genes clustered in the module, and the turquoise module clusters the most genes. The gene module analysis was performed with oil content as the sample trait, and the correlation coefficient between the pink module and the oil content reached a maximum of 0.94 (Figure 3B). Therefore, the pink module was mainly analyzed in-depth. The pink module clustered 345 genes, which were highly expressed in DAF90 (Figure 3C). The KEGG enrichment analysis found 345 genes were mainly enriched in pentose and glucuronate interconversions, metabolic pathways, fatty acid biosynthesis, carbon metabolism, and other important pathways (Figure 3D).

**Figure 4:**
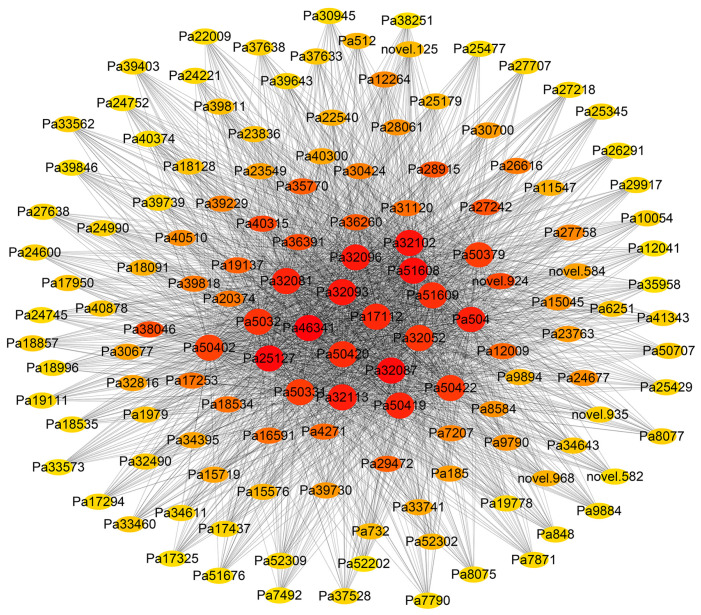
Co-expression network of top 150 genes. Note: Different network nodes in the graph each represent a gene, the color represents the edge weight of the gene’s adjacency matrix (representing the strength of the connection between two genes), and the larger the value, the darker the color, which represents that the two genes are closely connected or co-expressed; the shape size represents the degree of connectivity between the genes, where the larger the shape, the larger the degree of connectivity.

The visualization results of the top 150 genes are shown in Figure 4. The first-ranked hub gene was Pa46341, and the candidate genes with higher connectivity to hub genes were Pa25127, Pa51608, Pa51609, and Pa17112. The sequences of the candidate genes were compared on the NCBI website and named according to the name of the gene with the highest sequence repeat. Pa46341, Pa25127, Pa51608, Pa51609, and Pa17112 were named *DGAT1*, *LEC2*, *ABI3*, *ABI3*, and *GPAT5*, respectively.

### 3.4. The AKO Content and Fatty Acid Components

#### 3.4.1. Difference in the AKO Content

**Figure 5:**
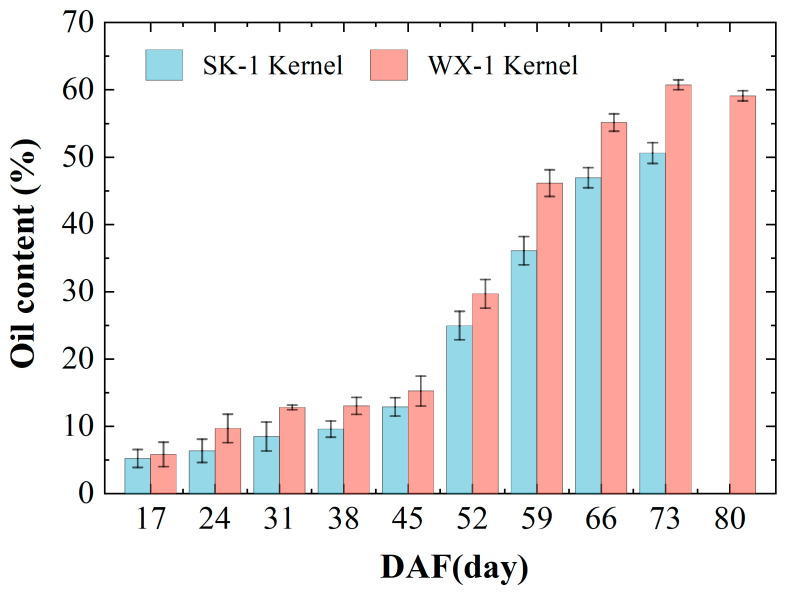
Patterns of apricot kernel oil accumulation in two varieties.

The AKO accumulation patterns of the two varieties were relatively similar (Figure 5); both showed a pattern of slow growth followed by rapid growth. During DAF17-38, the growth rates of ‘SK-1’ and ‘WX-1’ were 4.4% and 7.3%, respectively. During DAF38-73 was a period of rapid accumulation of oil, with growth rates of 41% and 47.7% for ‘SK-1’ and ‘WX-1’, respectively. At DAF73, the oil content of both varieties reached the maximum; the maximum oil content of ‘SK-1’ was 50.63%, and the maximum oil content of ‘WX-1’ was 59.1%. During DAF73-80, the oil content of ‘WX-1’ decreased by 1.63%, which may be related to the compositional transformation of apricot kernel contents at later stages of maturation. Multiple comparisons and analyses of variance were made with SPSS software, and the results (Table 2) showed that during the period of rapid oil accumulation (DAF38-DAF73), the difference in oil content among groups reached a significant level (*p* < 0.05).

#### 3.4.2. Differences in Fatty Acid Composition

**Figure 6:**
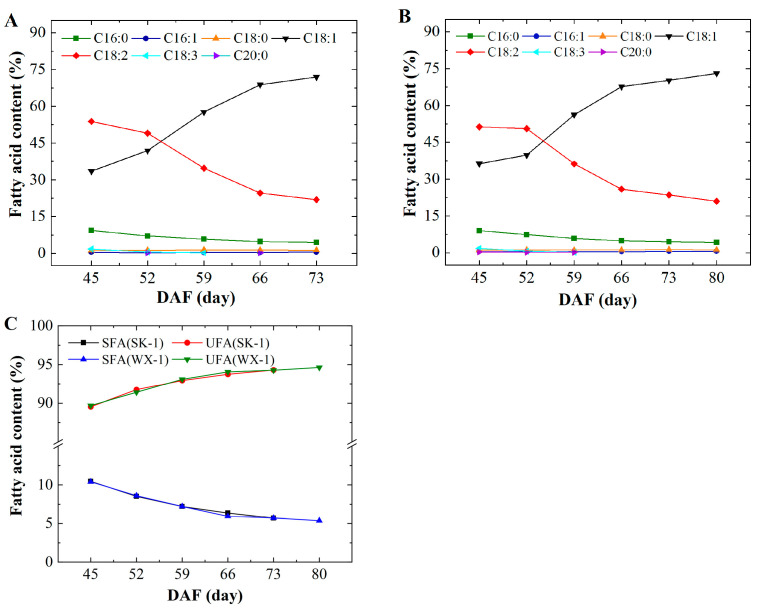
Changes in the fatty acid composition of the apricot kernel. (**A**) ‘SK-1’; (**B**) ‘WX-1’; (**C**) changes in saturated and unsaturated fatty acids.

The results of fatty acid determination showed that the fatty acid fractions of the two varieties were similar (Figure 6A,B). The fatty acid compositions of the apricot kernel were mainly C18:1, C18:0, and C16:0, with small amounts of C18:3, C18:2, C16:1, and C20:0. From DAF45 to DAF52, the content of C18:2 gradually decreased and C18:1 gradually increased. After DAF52, the content of C18:1 increased rapidly, and the total content was more than that of C18:2. The C18:1 content of both varieties reached the maximum value at the maturity stage. The content of C16:0 continued to decrease throughout the growth process. During the period of rapid lipid accumulation, unsaturated fatty acids (UFAs) were the major component, at more than 89% (see Figure 6C). At DAF42-80, the total content of saturated fatty acids (SFAs) decreased, and the content of UFA increased to 94%.

### 3.5. Expression Patterns of Candidate Genes for AKO Synthesis

**Figure 7:**
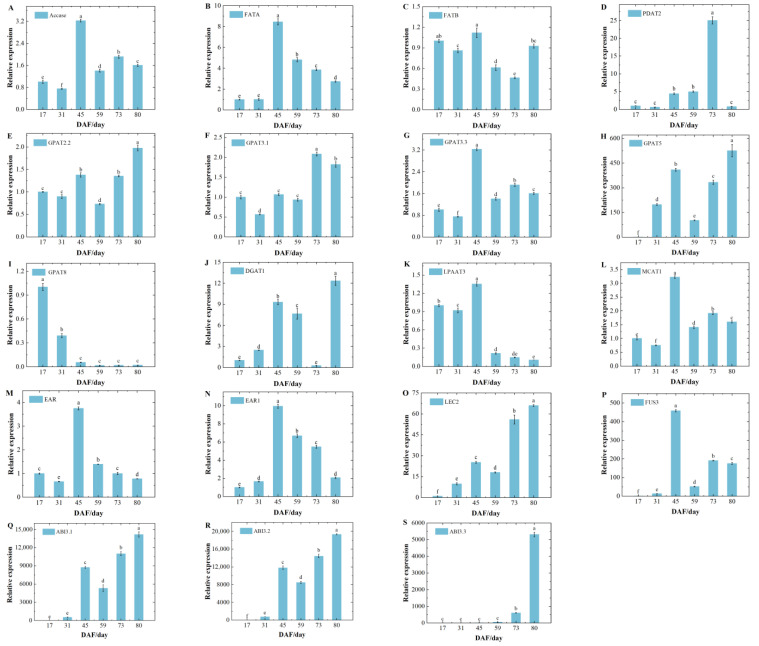
Relative expression of lipid synthesis-related genes in WX−1. Note: (**A**–**N**) enzyme genes, (**O**–**S**) transcription factors, and lowercase letters represent significant differences between groups (*p* < 0.05). Relative expression values, normalized to the *UBQ* gene, are shown as 2^−ΔΔCt^ relative to 17 DAF. Error bars represent the SD of three biological replicas with three technical replicas each. Different lowercase letters indicated significant difference between groups (*p* < 0.05).

**Figure 8:**
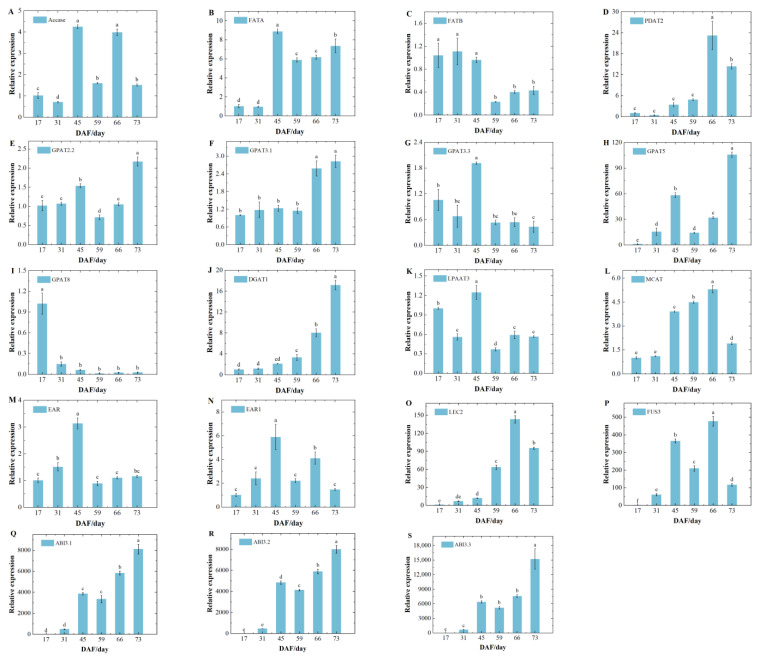
Relative expression of lipid synthesis-related genes in SK−1. (**A**–**N**) enzyme genes, and (**O**–**S**) transcription factors. Different lowercase letters indicated significant difference between groups (*p* < 0.05).

Candidate genes *ACTIN*, *CYP*, and *UBQ* for apricot reference genes were obtained from the literature [21]. Pre-experimental validation in two varieties showed that the expression of *UBQ* was relatively stable and superior to that of *CYP* and *ACTING* in all developmental periods. Therefore, the expression patterns of 14 enzyme genes and 5 transcription factors were characterized in this study using *UBQ* as the reference gene (Figure 7A–S and Figure 8A–S). The relative expression of *Accase*, *FATA*, *GPAT3.3*, *EAR*, and *EAR1* in the two varieties reached a maximum at DAF45 and decreased after DAF45. The relative expression of *FATB* for ‘WX-1’ showed a decreasing–increasing–decreasing–increasing trend (Figure 7C), reaching a maximum at DAF45. The relative expression of *FATB* for ‘SK-1’ was stably expressed during DAF17-45 (Figure 8C), rapidly decreased during DAF45-59, and then increased slightly. *PDAT2* showed similar behavior in both species (Figure 7D and Figure 8D), with a slow increase during DAF0-59, a sharp increase at DAF73, and then a sharp decrease. *GPAT2.2* and *GPAT5* showed an ‘N’-shaped (increasing–decreasing–increasing) trend and reached the maximum value in the last sampling period (Figure 7E,H and Figure 8E,H). The relative expression of *GPAT3.1* increased consistently, with ‘SK-1’ reaching a maximum at DAF73 and ‘WX-1’ decreasing slightly after reaching a maximum at DAF73. On the contrary, *GPAT8* showed a continuous rapid decrease. *DGAT1* showed the same trend as *GPAT3.1* in ‘SK-1’ with an ‘N’-shaped trend. *LPAAT3* showed an ‘increasing–decreasing–increasing’ trend in ‘SK-1’ and an increase then decrease in ‘WX-1’, reaching a maximum at DAF45. Transcription factor expression patterns were similar in the two varieties (Figure 7O–S and Figure 8O–S). The relative expression levels of transcription factors *ABI3.1*, *ABI3.2*, and *ABI3.3* exhibited a continuous upward trend, peaking at the last sampling. The trend of relative expression changes in *LEC2* in ‘WX-1’ closely resembled that of the ABI gene family, whereas in ‘SK-1’, it was continuously up-regulated at first and slightly decreased after DAF66. *FUS3* showed an ‘M’-shaped (increasing–decreasing–increasing–decreasing) trend in ‘SK-1’, reaching a maximum at DAF66. *FUS3* rose sharply during DAF31-45, fell sharply during DAF45-59, and rose slowly during DAF59-80.

### 3.6. Correlation Analysis

**Figure 9:**
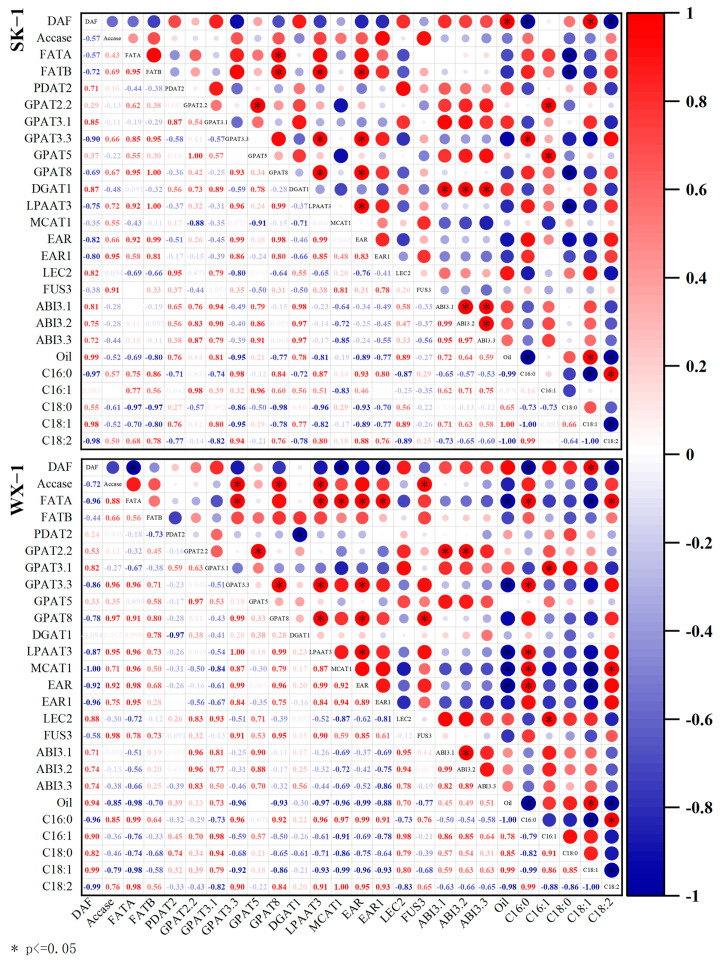
Correlation analysis between gene expression and lipid content.

To investigate the intrinsic relationships between AKO content, fatty acid composition, and gene expression, the correlation analysis was performed on two varieties (Figure 9). In ‘SK-1’, the genes highly correlated with oil content were *GPAT3.1* and *LEC2*, while the significantly correlated genes included *PDAT2*, *DGAT1*, and three members of the *ABI* family. In ‘WX-1’, the genes significantly associated with oil content were *GPAT3.1*, *LEC2*, and *ABI3.3*. In regulating fatty acid composition, the genes significantly correlated with C18:1 content included *GPAT3.1*, *LEC2*, *ABI3.1*, and *ABI3.2*; furthermore, the genes significantly correlated with ‘SK-1’ also comprised *PDAT2*, *DGAT1*, and *ABI3.3*. The genes significantly associated with C18:2 content included *Accase*, *FATA*, *FATB*, *GPAT3.3*, *GPAT8*, *LPAAT3*, *EAR,* and *EAR1*. Among these, *FATA*, *GPAT3.3*, *GPAT8*, *LPAAT3*, *EAR*, and *EAR1* exhibited a high correlation.

## 4. Discussion

### 4.1. Patterns of Oil Accumulation and Fatty Acid Composition in AKO

AKO can be used for edible oil and biodiesel processing as an excellent woody oilseed. Its high-value processing potential has attracted the attention of scholars at home and abroad. Apricot kernel’s different varieties and origins result in a wide range of oil contents, varying from 30% to 60% [22,23,24,25]. In this study, the accumulation of AKO in two varieties showed an ‘S’-shaped growth pattern with a slow increase followed by a sharp increase, similar to the results of Stryjecka [26]. In addition, the results demonstrated that apricot kernel can be harvested about 7 days before full ripeness to obtain the maximum oil content in order to maximize economic value.

The fatty acid composition of AKO is mainly C18:1 (more than 70%), followed by C18:2, and so on [27,28]. In this study, the two varieties at maturity have more than 70% of C18:1, more than 20% of C18:2, and greater than 94% of unsaturated fatty acids. However, at DAF30, the C18:1 content is only approximately 30%, whereas the C18:2 content is as high as 50%. This is attributed to the fact that the desaturation process strengthens the transformation of fatty acid composition in lipid accumulation. Furthermore, C18:1 has cholesterol-lowering properties [29], and C18:2 significantly reduces the risk of cardiovascular disease, cancer, diabetes, obesity, and other diseases [30]. It shows that AKO has great potential for development in the medical and healthcare fields [31,32].

### 4.2. Biosynthesis of Apricot Kernel Oil

**Figure 10:**
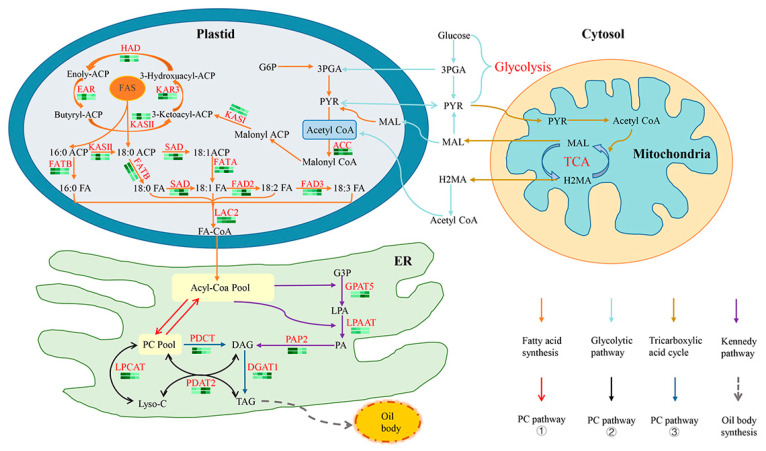
Biosynthesis of apricot kernel oil. Note: On the transcriptome of the apricot kernel, the icons below each enzyme ID show the expression patterns of the transcripts through each of the four seed developmental stages, with the color scale indicating the levels of expression of genes: deeper green indicates high expression and light green indicates lower expression. Above is the gene expression pattern for WX-1, and below is the gene expression pattern for SK-1. Expression patterns are shown only for genes that were found to have RPKM values of ≥2. Ketoacyl-ACP synthase, KAS; 3-ketoacyl-CoA reductase, KAR; ethylene response factor-associated amphiphilic repression, EAR; stearoyl-ACP desaturase, SAD; fatty acid acyl-ACP thioesterase, FAT; stearoyl-ACP desaturase, SAD; fatty acid desaturase, FAD; long-chain acyl-coenzyme A synthetase LACS; glycerol-3-phosphate acyltransferase, GPAT; lysophosphatidic acid acyltransferase, LPAAT; phosphatidic acid phosphatase, PAP; phosphatidylcholine: diacylglycerol cholinephosphotransferase, PDCT; diacylglycerol acyltransferase, DGAT; lysophosphatidylcholine acyltransferase, LPCAT; phospholipid: diacylglycerol acyltransferase, PDAT; phosphatidylcholine, PC; glycerol-3-phosphate, G3P.

In this study, two varieties of almonds, with a low oil content (‘SK-1’) and high oil content (‘WX-1’), were used as materials. A molecular model for the synthesis of AKO is proposed based on the results of this study (Figure 10). The biosynthesis of AKO consists of three main processes: (1) fatty acid synthesis and modification, (2) TAG synthesis, and (3) oil body formation. The initial substrate for fatty acid synthesis is acetyl-CoA. Acetyl-CoA in plastids is catalyzed by ACCase, the primary rate-limiting enzyme for fatty acid synthesis, to synthesize malonyl CoA, which is then acylated to form malonyl ACP before entering the carbon chain extension reaction. The carbon chain extension is catalyzed sequentially by KAR4, HAD, EAR, and KAS II enzymes, completing the extension of one carbon chain with an increase of two carbon atoms per cycle. A 16:0 ACP is formed after six cycles, and an 18:0 ACP is formed after seven cycles; 16:0 ACP and 18:0 ACP are catalyzed by FATB enzyme to form 16:0 FA and 18:0 FA; 18:0 ACP is desaturated by the SAD enzyme to form 18:1 ACP, which is further catalyzed by the FATA enzyme to form 18:1FA; 18:0FA is catalyzed by SAD, FAD2, and FAD3 enzymes to form 18:1FA, 18:2FA, and 18:3FA sequentially; and, finally, all free fatty acids are catalyzed by the LEC2 enzyme to form FA-CoA [33].

In the endoplasmic reticulum (ER), FA-CoA from plasmodesmata enters the Acyl-Coa pool. There are two pathways for TAG synthesis. One is the Kennedy pathway (Figure 10 purple arrows): G3P and FA-CoA undergo three acylation reactions catalyzed by GPAT3, LPAAT, and PAP2 to form DAG, which DGAT1 catalyzes to synthesize TAG [34]. The other is the flow of fat through the PC to eventually synthesize TAG [32]. There are three branches in this pathway: (1) The primary fatty acids from plastids are released into the Acyl-Coa Pool after acyl modifications are completed on the PC (Figure 10 red arrows) and continue into the Kennedy pathway. (2) The direct transfer of FA into PC to DAG, which is then catalyzed by PDAT2 to synthesize TAG (black arrow in Figure 10), overlaps with the last step of the Kennedy pathway reaction. (3) The synthesis of TAG by DAG from PC is catalyzed by DGAT1 (Figure 10 blue arrow). TAG is stored in the oil body as a TAG–protein complex in the cytoplasmic matrix after binding to oil body proteins [35].

In the fatty acid synthesis pathway, the expression changes in enzyme genes involved in carbon chain elongation reactions are similar between the two varieties. During the desaturation process, the expression levels of the *SAD*, *FATA*, *FATB*, and *FAD2* genes are up-regulated during the rapid accumulation of lipids, which accounts for the similarity in the primary components of almond oil. In the synthesis of TAG, ‘WX-1’ depends not only on the Kennedy pathway but also significantly on the PC pathway regulated by *PDAT2*. The expression level of *GPAT3* is significantly up-regulated during the rapid accumulation of oil in ‘SK-1’, and the expression level of the key regulatory enzyme *PDAT2* in the PC pathway is lower than that of ‘WX-1’. The results indicate that the synthesis of TAG in ‘SK-1’ primarily relies on the Kennedy pathway, with a lesser contribution from the PC pathway. DGAT1 is the last key enzyme in TAG synthesis, and its expression level during the oil rapid accumulation of ‘WX-1’ is greater than ‘SK-1’. Consequently, the AKO content in mature ‘WX-1’ surpasses that of ‘SK-1’. This finding offers a significant molecular foundation for understanding the biosynthesis of AKO.

### 4.3. Key Genes of AKO Biosynthesis

During fatty acid synthesis, ACCase catalyzes the formation of malonyl-CoA from acetyl-CoA, which is the key step for acetyl-CoA to enter the fatty acid synthesis pathway. The relative expression of the *IpACCase* gene in 130 DAT santolina is significantly higher than that in 70 DAT [36]. The relative expression of *ACCase* in this study increases rapidly from DAF31-45, reaches a maximum at DAF45, and shows a decreasing trend after DAF45, which is consistent with the results in peony seed oil [37]. AKO accumulates gradually during early development, with lipid content starting to rise significantly after DAF30. This increase necessitates more *ACCase* enzymes to ensure that an adequate amount of precursor acetyl-CoA enters the fatty acid synthesis pathway. The rapid up-regulation of the relative expression of *ACCase* preceded the period of rapid lipid accumulation. This indicates a lag effect of changes in substance synthesis relative to changes in gene expression. The decrease in expression after DAF45 was due to the feedback of lipid accumulation, which inhibited the expression of the *ACCase* gene. In the later period, the oil content increased steadily, and the expression of *ACCase* was relatively stable.

FATA and FATB are two subfamilies of FAT that play important roles in regulating the carbon chain length and components of fatty acids [38]. FATA catalyzes the synthesis of unsaturated fatty acids, with C18:1 being the preferred substrate. FATB positively regulates the levels of saturated fatty acids, primarily facilitating the synthesis of 16:0 and 18:0 [39]. In both varieties of apricot kernel, the relative expression of *FATA* increases significantly during the period of rapid lipid accumulation, and both are higher than the expression of *FATB*, which is consistent with the result that oleic acid is the major fatty acid (Figure 7B,C and Figure 8B,C). GPAT is the key rate-limiting enzyme for the first step of the acylation reaction in TAG synthesis, catalyzing the acyl transfer on Acyl Co-A to the sn-1 position of G3P to convert to LPA. *BnGPAT4* regulates the total lipid content and fatty acid composition in oilseed rape [40]. In this study, five genes of the *GPAT* family showed different expression patterns. *GPAT2.2*, *GPAT3.1,* and *GPAT5* were up-regulated during the period of rapid lipid accumulation in both varieties. *GPAT8* was consistently down-regulated during the process of lipid accumulation. *GPAT3.3* was significantly up-regulated during the period of rapid lipid accumulation, but its expression declined with the accumulation of lipids. This suggests that the *GPAT2.2*, *GPAT3.1*, and *GPAT5* genes promote lipid synthesis and can be used as candidate genes for later functional validation. DGAT is the enzyme that catalyzes the acylation of acyl-coenzyme A and DAG at the n-3 position in the Kennedy pathway [41]. PDAT catalyzes the transfer of fatty acyl groups from the sn-2 site of PC to the sn-3 site of diacylglycerol to form TAG [42]. PDAT1 has an overlapping function with DGAT1 in TAG biosynthesis in *arabidopsis* seeds [43].

A similar complementary relationship exists between *PDAT1* and *DGAT1* in the apricot kernel. The relative expression of *PDAT2* in ‘WX-1’ grows slowly from DAF0-59 (Figure 7D), but the relative expression of *DGAT1* increases dramatically during the same period (Figure 7J). At DAF73, the relative expression of *PDAT2* increases rapidly and reaches the maximum value, and the expression of *DGAT1* decreases rapidly to the minimum value. At DAF80, the relative expression of *PDAT2* decreases rapidly but the expression of *DGAT1* increases rapidly to the maximum value, which is related to the substrate preference of the two enzymes. Different TAG synthesis pathways contribute differently to lipid accumulation at various stages, and this synchronized complementary mechanism provides important insights for addressing the issue of oil synthesis. The expression pattern of *PDAT2* is the same in the two varieties (Figure 7D and Figure 8D), but the *DGAT1* of ‘SK-1’ (Figure 8J) does not show a trend of synchronous complementarity; instead, it exhibits a similar trend to that of *PDAT2*. This indicates that there is varietal variability in the complementation function between these two genes, and it is also possible that there are other pathways in ‘SK-1’ TAG synthesis, which needs to be further investigated.

In this study, all five transcription factors were highly expressed during the lipid accumulation phase (Figure 7O–S and Figure 8O–S). Among them, the expression levels of *LEC2* and *ABI3.3* were higher in ‘SK-1’, whereas those of *ABI3.1* and *ABI3.2* were higher in ‘WX-1’. The expression of *FUS3* varies at different developmental periods, indicating that the sensitivity of *ABI3* and *FUS3* to *LEC2* activation is different in different varieties. The expression changes in the three *ABI3* genes in ‘WX-1’ are consistent with that of *FUS3*. At DAF45, the expression trends of *ABI3.1* and *ABI3.2* in ‘SK-1’ are consistent with that of *FUS3*, and all of them increase rapidly to reach the peak. However, the *ABI3.3* remains at a low level. During the maturation stage, the expression of *FUS3* decreases, but the expression of the three *ABI3* family genes increases rapidly. This may be due to the high activation strength of *ABI3* genes by *FUS3* within a particular range. When the expression of *FUS3* exceeds a certain threshold value, its activation effect begins to weaken.

## 5. Conclusions

This study aimed to identify the key genes that regulate AKO synthesis and fatty acid composition, as well as analyze the molecular mechanisms underlying oil accumulation. This research examined the oil content and fatty acid profiles of two varieties of apricot kernels, utilizing completed transcriptome sequencing, WGCNA, and qRT-PCR correlation analysis of 24 samples. Based on the results and discussion, some main conclusions can be drawn as follows:

(1) The oil accumulation of apricot kernel shows an ‘S’-type accumulation pattern, and the fatty acid fractions in the maturity stage are mainly oleic acid and linoleic acid, which can be converted into each other.
(2) Transcriptome sequencing was analyzed to obtain 17,411 differentially expressed genes. These were screened and analyzed by WGCNA and divided into seven modules. Five Hub genes were obtained by constructing a co-expression network in the pink module.
(3) The significant differential expression of three key enzymes for fatty acid synthesis (*Accase*, *FATA*, and *FATB*), three key enzymes for TAG synthesis (*PDAT2*, *GPAT5*, and *DGAT1*), and three families of transcription factors (*LEC2*, *FUS3*, and *ABI3*) was screened and verified.

The results offer a theoretical foundation for further elucidating the molecular mechanisms underlying AKO synthesis and accumulation. Additionally, they also provide potential functional genes for enhancing the AKO content and artificially regulating fatty acid fractions. The functional verification of these genes, along with the exploration of upstream and downstream gene interaction mechanisms, represent new directions for future research.

## Figures and Tables

**Table 1:** qRT-PCR primer sequences of candidate genes.

Gene Name	Primer-S	Primer-A
*ACCase*	ATCAAGGCAACGGCAGGA	CCGCTCTCCAAAGTGAACAAC
*FATA*	GGAAGAGCAACAAGTAAGTGGGT	CTATTGTTAGGCTCTGGAAAGGC
*FATB*	GTCTTCCGCCAAAACTTCTCA	ATCTCCCAATAGCCCAGCAG
*PDAT2*	CAAGTGAAAGAACCTGTGAAGTATG	TCTGATGCTTTGCCTGCTTAG
*GPAT2.2*	TGGACAAACACACAGTAGAGCA	AGTAGAGGAACTTTAGAGCACCC
*GPAT3.1*	ATCCAACAAGAACCGCACG	ACACCACAAAGGGAACAAATACAC
*GPAT3.3*	TTGTGTTACTCTGGTCCTGCC	GGTTTGGTCTTGTGGGGTTA
*GPAT5*	ACCCTGACCAACCAAAAGCA	CCAAGACTATCCGAATGAAGGC
*GPAT8*	CTGACGCTGCCCGCTTAT	TGCAGTTGACCGCCACG
*DGAT1*	GATGCCGTGCCCAGTTC	AACCCTTTCGAACACATGCT
*LPAAT3*	GTTACTCTGGTCCTGCCTACTTG	TCCTTGGTTTGGTCTTGTGG
*MCAT*	CTGTATCTGGTGGTGTGAAAGGA	TTCTTGGTGTTCTGATCTGTGTTG
*EAR*	AGGGCATTTATCGCTGGTGT	TGTTCAGAGCAGGCACCCA
*EAR-1*	TCTCACATCTCAGCAACACAGC	TCAATAGGCAATCCTGGCAA
*LEC2*	AGTTGGAGGTGGGAGATTGC	GTGTTGGCGTCAGCGTAGTTA
*FUS3*	CCCACAATGGCAATGGATGA	AGAGTCATTTGAGAAGGTGGTGGT
*ABI3-1*	CGAGGAGTGAAGGTACGGCA	CTGAGACTGAGGAAGGTGACGAA
*ABI3-2*	GATGACGACCACCAGCAATTA	TGACCTCAGCCACTCCAAGA
*ABI3-3*	CACTTCCCTCCTCTCCCTGAT	CGCATCAACCGCCTTATCA

Note: acetyl-CoA carboxylase, ACCase; fatty acyl carrier protein thioesterase A, FATA; fatty acyl carrier protein thioesterase B, FATB; phospholipid: diacylglycerol acyltransferase, PDAT; glycerol-3-phosphate acyltransferase, GPAT; diacylglycerol acyltransferase, DGAT; lysophosphatidic acid acyltransferase, LPAAT; malonyl-coen-zyme A-acyl carrier protein transacylase, MCAT; ethylene response factor-associated amphiphilic repression, EAR; leafy cotyledon 2, LEC2; Fusca3, FUS3; abscisic acid insensitive 3, ABI3.

**Table 2:** Multiple comparisons of oil content of apricot kernel.

Day After Flowering	Oil Content (Average ± Standard Error)	
	WX-1	SK-1
17	0.0583 ± 0.0105 g	0.0524 ± 0.0076 h
24	0.0972 ± 0.0122 f	0.0638 ± 0.001 gh
31	0.1284 ± 0.0021 e	0.085 ± 0.0124 fg
38	0.1306 ± 0.0073 e	0.0961 ± 0.0069 f
45	0.1527 ± 0.0128 e	0.129 ± 0.0079 e
52	0.2969 ± 0.0123 d	0.249 ± 0.012 d
59	0.4616 ± 0.0115 c	0.361 ± 0.0121 c
66	0.5517 ± 0.0074 b	0.4697 ± 0.0087 b
73	0.6074 ± 0.0042 a	0.5063 ± 0.0089 a
80	0.5911 ± 0.0043 a	

Note: Different lowercase letters in the same column indicate significant differences among different days after flowering (*p* < 0.05).

## Data Availability

The original contributions presented in this study are included in the article. Further inquiries can be directed to the corresponding author.
